# Targeting phytoprotection in the COVID-19-induced lung damage and associated systemic effects—the evidence-based 3PM proposition to mitigate individual risks

**DOI:** 10.1007/s13167-021-00249-y

**Published:** 2021-08-03

**Authors:** Alena Liskova, Lenka Koklesova, Marek Samec, Basma Abdellatif, Kevin Zhai, Manaal Siddiqui, Miroslava Šudomová, Sherif T.S. Hassan, Erik Kudela, Kamil Biringer, Frank A. Giordano, Dietrich Büsselberg, Olga Golubnitschaja, Peter Kubatka

**Affiliations:** 1 Department of Obstetrics and Gynecology, Jessenius Faculty of Medicine, Comenius University in Bratislava, 03601 Martin, Slovakia; 2 Weill Cornell Medicine-Qatar, Education City, Qatar Foundation, Doha, 24144 Qatar; 3 Museum of Literature in Moravia, Klášter 1, 66461, Rajhrad, Czech Republic; 4 Department of Applied Ecology, Faculty of Environmental Sciences, Czech University of Life Sciences Prague, Kamýcká 129, 16500 Prague, Czech Republic; 5 Department of Radiation Oncology, University Hospital Bonn, Rheinische Friedrich-Wilhelms-Universität, Bonn, Germany; 6 Predictive, Preventive and Personalised (3P) Medicine, Department of Radiation Oncology, University Hospital Bonn, Rheinische Friedrich-Wilhelms-Universität Bonn, 53127 Bonn, Germany; 7 Department of Medical Biology, Jessenius Faculty of Medicine, Comenius University in Bratislava, 03601 Martin, Slovakia

**Keywords:** Phytochemicals, Phenolic compounds, Phenolic acids, Flavonoids, Coumarins, Stilbenoids, Inflammation, Immunity, Cytokine storm, Lung damage, ARDS, Predictive preventive personalized medicine (3PM/PPPM), Anti-inflammation, Antibacterial, Antiviral, COVID-19, Cancer, Chronic diseases, Risk assessment, Signaling pathways, Therapy efficacy, Disease management, Health economy, Health policy

## Abstract

The risks related to the COVID-19 are multi-faceted including but by far not restricted to the following: direct health risks by poorly understood effects of COVID-19 infection, overloaded capacities of healthcare units, restricted and slowed down care of patients with non-communicable disorders such as cancer, neurologic and cardiovascular pathologies, among others; social risks—restricted and broken social contacts, isolation, professional disruption, explosion of aggression in the society, violence in the familial environment; mental risks—loneliness, helplessness, defenceless, depressions; and economic risks—slowed down industrial productivity, broken delivery chains, unemployment, bankrupted SMEs, inflation, decreased capacity of the state to perform socially important programs and to support socio-economically weak subgroups in the population. Directly or indirectly, the above listed risks will get reflected in a healthcare occupation and workload which is a tremendous long-term challenge for the healthcare capacity and robustness. The article does not pretend to provide solutions for all kind of health risks. However, it aims to present the scientific evidence of great clinical utility for primary, secondary, and tertiary care to protect affected individuals in a cost-effective manner. To this end, due to pronounced antimicrobial, antioxidant, anti-inflammatory, and antiviral properties, naturally occurring plant substances are capable to protect affected individuals against COVID-19-associated life-threatening complications such as lung damage. Furthermore, they can be highly effective, if being applied to secondary and tertiary care of noncommunicable diseases under pandemic condition. Thus, the stratification of patients evaluating specific health conditions such as sleep quality, periodontitis, smoking, chronic inflammation and diseases, metabolic disorders and obesity, vascular dysfunction, and cancers would enable effective managemenet of COVID-19-associated complications in primary, secondary, and tertiary care in the context of predictive, preventive, and personalized medicine (3PM).

## Introduction

### Risks related to the COVID-19 pandemic conditions with consequences for healthcare

The risks related to the COVID-19 are multifaceted including but by far not restricted to the following:

- Direct health risks by poorly understood effects of COVID-19 infection, overloaded capacities of healthcare units, restricted and slowed down care of patients with non-communicable disorders such as cancer, neurologic and cardio-vascular pathologies, amongst others;
- Social risks—restricted and broken social contacts, isolation, professional disruption, explosion of aggression in the society, violence in the familial environment;
- Mental risks—loneliness, helplessness, defenceless, depressions;
- Economic risks—slowed down industrial productivity, broken delivery chains, unemployment, bankrupted SMEs, inflation, decreased capacity of the state to perform socially important programs and to support socio-economically weak subgroups in the population.

Directly or indirectly, the above listed risks will get reflected in a healthcare occupation and workload that is a tremendous long-term challenge for the healthcare capacity and robustness. Indeed, in a long-term way a significant increase in the incidence of mental disorders can be expected as well as generally increased mortality of noncommunicable diseases, due to the late diagnoses and delayed treatments.

### What did we learn from the COVID-19 pandemic in healthcare?

Enormous long-term socioeconomic burden caused by the COVID-19 pandemic is currently resulting in the global acknowledgment of prediction, prevention, and personalization in healthcare as an unavoidable approach to save lives and to protect societies in a cost-effective manner.

Due to limited care capacities that medical units possess under pandemic conditions, per evidence, chronically diseased patients demonstrate poorer live-quality and outcomes, and consequently increased mortality rates [1]. Overall management of severe chronic diseases such as cancer is currently more challenging than in general. In this condition, healthcare givers are prompted to consider pandemic-related risks additionally to conventional ones in the area. Personalized risk assessment is an essential pillar of predictive strategies and targeted prevention in cancer management [2].

Comorbidities significantly increase the risks of poor outcomes in COVID-19-infected individuals. In particular, inflammation-related pathologies such as cancer are relevant in a reciprocal manner. To this end, the severe COVID-19 course is characterized by hyper-cytokinemia, an exaggerated immune response, and excessive release of pro-inflammatory cytokine mediators, called “cytokine storm.” Affected individuals, even with mild disease course, months after the infection exhibit diffuse multiorgan symptoms similar to those reported for children with *Multisystem Inflammatory Syndrome* [3]. Moreover, the study performed in the UK demonstrated 80% of COVID-19-infected patients were treated at intensive care units, and over 50% mortality was associated with bacterial superinfections and severe disease course [4]. Proposed pathomechanisms consider evident interactions between the viral particles and the host microbiota including the oral cavity, the respiratory and gastrointestinal tracts [5].

Expert recommendations consider:

- optimal oral hygiene crucial for improved individual outcomes and reduced morbidity under the COVID-19 pandemic condition,
- anti-inflammatory medication,
- dual anti-microbial and anti-viral therapeutic effects as particularly effective for tertiary care to avoid severe complications linked to COVID-19 [4, 6–8].

### Approaches mitigating individual health risks are highly requested under pandemic and post-pandemic conditions

The article does not pretend to provide solutions for all kind of health risks. However, it aims to present the scientific evidence of great clinical utility for primary, secondary, and tertiary care to protect affected individuals in a cost-effective manner. To this end, due to below discussed antimicrobial, antioxidant, anti-inflammatory, and antiviral properties, naturally occurring plant substances are capable to function as anti-COVID-19 agents also protecting affected individuals against COVID-19-associated life-threatening complications such as lung damage. Furthermore, they can be highly effective, if being applied to secondary and tertiary care of noncommunicable diseases under pandemic condition. Thus, the stratification of patients evaluating specific health conditions such as sleep quality [9], periodontitis [10], smoking [11], chronic diseases or chronic inflammation [12, 13], metabolic disorders or obesity [14], vascular dysfunction [15, 16], or cancer [17] would enable effective managemenet of COVID-19-associated complications in primary, secondary, and tertiary care in the context of 3PM. Indeed, flavonoids are abundant compounds found in many plants that are synthesized in a response to microbial attacks and are therefore expected to possess antimicrobial and antiviral capacity [18]. Also, phenolics are potent anti-inflammatory agents [19]. Therefore, phenolics exert significant mitigating effects in tertiary cancer care associated with their potent anti-inflammatory, antimicrobial, and antiviral activities.

#### Antiviral effects

Plant phenolics are associated with a significant antiviral capacity [20–25]. Indeed, numerous phenolic compounds exert potent antiviral capacity against the coronavirus family including novel SARS-CoV-2 virus [18, 26–29]. In addition, specific viruses (hepatitis B, hepatitis C, human papillomavirus, and human oncogenic herpesviruses such as Epstein–Barr virus and Kaposi’s sarcoma-associated herpesvirus) contribute to about 10 – 15 % global burden of human cancers [30–32]. Therefore, the utilization of antiviral compounds, such as naturally occurring flavonoids or other phenolics, to eliminate or suppress viral infections may significantly improve cancer management [30, 33, 34].

#### Anti-inflammatory effects

High morbidity and mortality associated with COVID-19 are related to the release of pro-inflammatory cytokines and thrombogenic agents leading to the destruction of the lung. However, many patients who recovered from or had mild COVID-19 symptoms exhibit symptoms of multisystem inflammatory syndrome originally reported in children, and similar conditions are observed in adults [3]. To this end, phenolics are highly effective against inflammatory storm [19] and multisystem inflammatory symptoms [3, 35–38] and thus improving the overall outcomes of COVID-19 patients also in the context of potential tertiary cancer care. Indeed, the potent anti-inflammatory capacity of phenolics offers novel opportunities to improve overall cancer management [39].

#### Antimicrobial protection

Plant phenolics are well-known antibacterial agents while the proposed antibacterial mechanisms include the inhibition of nucleic acid synthesis, inhibition of cytoplasmic membrane function, inhibition of the attachment and biofilm formation, inhibition of the porin on the cell membrane, inhibition of energy metabolism, alteration of the membrane permeability, and attenuation of the pathogenicity [40–43]. Pathologic composition of the gut microbiome can lead to the accumulation of pro-inflammatory and pro-tumorigenic bacteria or depletion of protective bacteria and eventually leads to colorectal cancer risk. Nevertheless, phenolics, especially flavonoids, exert a potent capacity to modify the gut microbiome, inflammatory responses, and the risk of colorectal cancer [44–46]. Further, flavonoids demonstrate periodontic benefits demonstrated through anti-microbial and anti-inflammatory efficacy [47].

## COVID-19-associated lung injury

Human-to-human transmission of SARS-CoV-2 is mediated mainly through droplets emitted by coughing and sneezing [48, 49]. The specific adaptive immune response is essential to eliminate the virus and avoid its progression from the asymptomatic initial stage. However, impairment of the immune response allows the infection to propagate, leading to tissue damage and inflammation. Lung inflammation is considered a major cause of life-threatening respiratory events in severe cases of COVID-19 [49]. Acute lung injury (ALI) is associated with severe inflammatory reactions resulting in the deterioration of gas exchange while lung edema as one of ALI manifestations can eventually lead to acute respiratory distress syndrome (ARDS) [50]. COVID-19-associated ARDS primarily results from a deregulated host response, followed by damage of alveolar cells and lung fibrosis. Exaggerated release of pro-inflammatory cytokines (cytokine storm) and loss of T lymphocytes describes the most aggressive presentation [51].

ARDS is characterized by acute and diffuse inflammatory damage of the alveolar-capillary barrier related to increased vascular permeability and reduced lung size and compliance while these factors compromise gas exchange [52]. COVID-19 is an acute respiratory disease, and nearly one third of COVID-19 patients develop severe lung edema, dyspnea, hypoxemia, or ARDS and more than half of patients with ARDS die [50]. ARDS is histopathologically defined as diffuse alveolar damage (DAD) of the lung [52]. DAD progresses through stages—the early acute or exudative phase, the proliferative or organizing phase, and the fibrotic phase [48, 53]. Autopsies of most SARS-CoV-2 patients revealed different stages of DAD with fibrin-rich hyaline membranes, a high frequency of macro- and microvascular thrombosis [54, 55], interstitial and intra-alveolar proteinaceous edema, and type II pneumocyte hyperplasia, all of which are consistent with the ARDS histopathologic background [56]. Despite primary SARS-CoV-2 infection, DAD can be promoted by secondary infections such as bronchopneumonia and aspergillosis [48]. The action of SARS-CoV-2 in lung cells and ARDS-induced deregulation of immune-coagulative pathways affect the extent of pulmonary involvement in severe COVID-19.

Moreover, autopsies confirmed pulmonary fibrosis as a common event in COVID-19 [54]. Therefore, mortality in COVID-19 is strongly related to DAD and associated immunothrombosis in pulmonary capillary networks and adjacent vessels. DAD, in turn, is linked to SARS-CoV-2 infection of pneumocytes and endothelial cells, dysfunction of the pulmonary vasculature and systemic endothelial tissue, and associated aberrant cytokine responses [57]. Besides typical DAD, acute fibrinous and organizing pneumonia (AFOP) was reported in postmortem biopsies of COVID-19 patients [48]. In addition to ARDS-associated features of diffuse alveolar injury observed in ARDS, fibrin thrombosis is often observed in the small pulmonary vasculature of COVID-19 patients; therefore, microthrombic complications contribute to the progression of COVID-19 [58]. Indeed, microthrombi were found in autopsies of COVID-19 patients with fibrin deposition within the capillaries. Microthrombi were also found in patients with less pronounced DAD [48].

**Fig. 1:**
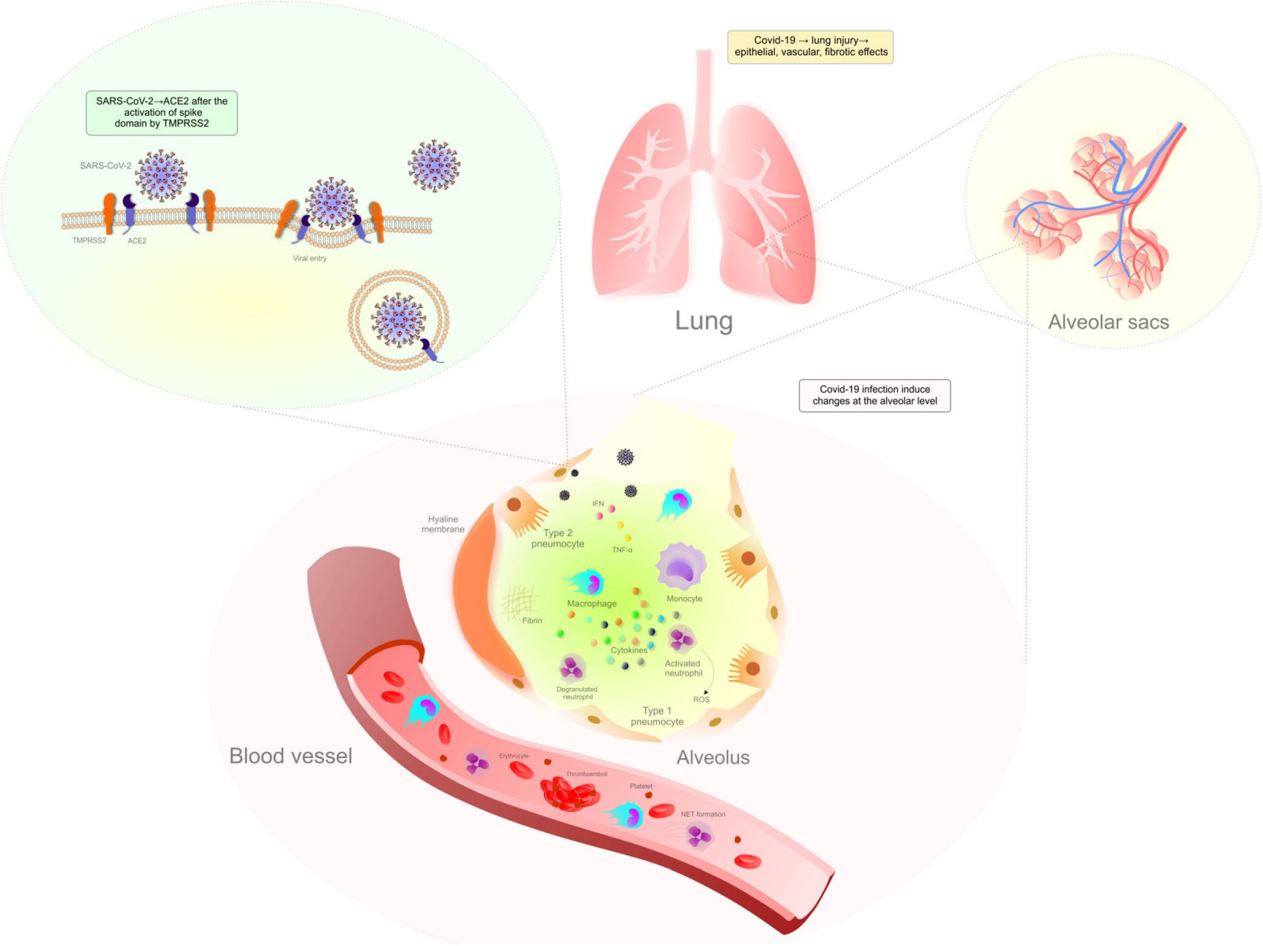
COVID-19: lung injury comprises epithelial, vascular, and fibrotic effects. Abbreviations: ACE2, angiotensin-converting enzyme 2; AFOP, acute fibrinous and organizing pneumonia; DAD, diffuse alveolar damage; IFN, interferon; NET, neutrophil extracellular traps; ROS, reactive oxygen species; TMPRSS2, transmembrane serine protease 2; TNF-α, tumor-necrosis factor-alpha; →, leading to/resulting in. Explanatory notes: SARS-CoV-2 enters the cell through the ACE2 receptor after the activation of the spike domain by TMPRSS2 [48, 49]. Lung injury by COVID-19 has epithelial, vascular, and fibrotic effects [48]. Pulmonary alveoli are lined with type I and II epithelial cells surrounded by capillaries to promote gas exchange. SARS-CoV-2-induced alveolar damage → build-up of debris and fluid inside the alveolus (affects gas exchange associated with pro-inflammatory responses, increases vascular permeability, and induces fibrosis with collagen deposition and thrombosis) [48]. SARS-CoV-2 directly infects alveolar type I and II epithelial cells → loss of epithelial layer and occurrence of DAD (stimulated by the innate immune response). Alveolar capillary damage → fluid accumulation (capillary leak and intra-alveolar hemorrhage). DAD: *exudative* → hyaline membranes of serum proteins and condensed fibrin [48, 53], interstitial and alveolar edema, capillary congestion and microthrombi, inflammatory cell infiltrates, scattered fibroblasts, thickening of the alveolar septa, denudation of the alveolar epithelium, and type II hyperplasia within a few days of infection; *organizing* → proliferation of fibroblasts in septa and alveolar spaces to begin tissue repair through the organization of edema, the disappearance of hyaline membranes, atypia of pneumocytes, thromboembolic in capillaries and pulmonary vessels; *fibrotic* → stage in approximately 3 to 4 weeks [48]. Intra-alveolar hemorrhage is also observed in COVID-19 patients. The alveolar space can be infiltrated by inflammatory cells, neutrophils, megakaryocytes, macrophages, and other components that damage its function [48]. Monocytes recruited into the alveolar space secrete pro-inflammatory cytokines and induce the apoptosis of pneumocytes through the release of IFN through a pathway dependent on TNF-α activating cell death receptors. Macrophages release chemokines and other cytokines → increased permeability and the recruitment of neutrophils. Excessive neutrophil degranulation leads to damage and breakage of the alveolar-capillary barrier [52]. AFOP → formation of fibrin balls in the alveolar space blocks airflow into the alveoli, formation of hyaline membrane, and thickening of the septa for gas exchange and can also contribute to fibrosis. Loss of type I and II epithelial cells contributes to the flooding of alveoli with blood, edema, or cellular debris due to lost or impaired ion channels; this further degrades epithelial cells and the surfactant layer (reduced area of gas exchange). In response, the surrounding alveoli increase in volume, and the forces on the alveolar walls increase leading to increased stress surrounding the foci of injury, leading to further damage. With the progress of lung injury, more alveoli are damaged which leads to decreased lung compliance. Indeed, alveolar collapse, surfactant dysfunction, intra-alveolar edema, lung inflammation, and focal fibrotic remodeling are all lung stress concentrators that cause further injury progression. Microthrombi were found in autopsies of COVID-19 patients with observed deposition of fibrin within the capillaries. Neutrophils also contribute to capillary damage through NETs, which promote vascular occlusion due to the high neutrophil content of clots in capillaries of the alveolar septa. Platelet, fibrinogen, and neutrophil microthrombi and NET formation associated with immunothrombosis were observed in COVID-19 patients’ lungs when compared with non-COVID-19 controls. Neutrophils generate excessive ROS, which induces further lung damage [48]

In conclusion, the pathogenesis of lung injury associated with COVID-19 is evident at the alveolar level and comprises epithelial, vascular, and fibrotic effects (Fig. 1).

## Inflammation and cytokine storm in COVID-19

After the recognition of viral antigens, innate and adaptive immunity is activated resulting in the production of proinflammatory cytokines and chemokines. Although cytokine release is crucial for the defense against viral infection, aberrant immune responses can result in organ injury, especially in the elderly or comorbid patients. Therefore, immune activation can become so excessive that it causes uncontrolled systemic inflammatory responses, a process known as a cytokine storm. Such exaggerated immune response is demonstrated by the overproduction of proinflammatory cytokines and chemokines such as IL-6, IL-1β, tumor necrosis factor-alpha (TNF-α), GM-CSF, and IFN-γ, among others. The cytokine storm is associated with severe COVID-19 and unfavorable outcomes in hospitalized patients [19, 59, 60]. Moreover, plasma levels of IL-6, IL-8, and TNF-α peaked before death as demonstrated in autopsies of COVID-19 patients [59]. The cytokine storm can cause thrombotic events, ARDS, and multiple organ failure, while cytokine storm-induced damage predominantly in the lungs is considered a direct cause of death from acute COVID-19 [19].

Although specific molecular mechanisms are not fully understood, molecular mediators of inflammatory and immunologic pathways associated with COVID-19 pathogenesis and lung damage are detailed in Table 1.

**Table 1:** Molecular mediators of inflammatory and immunologic pathways associated with COVID-19 pathogenesis and lung damage

Molecular mediators	Mechanisms	Reference
NF-κB	Modulation of immune cell functions and cytokine expression in response to pathogenic stimuli. Many proinflammatory factors induce NF-κB signaling. NF-κB potentiates ROS production (leading to apoptosis in various tissues in diseases and viral infections). COVID-19 activation of NF-κB leads to the production of IL-1, IL-2, IL-6, IL-12, TNF-α, and GM-CSF.	[61, 62]
MMPs	MMP2 and MMP8 are upregulated in COVID-19-affected lung tissue. MMP9 released from neutrophils in ALI promotes inflammation and degradation of the alveolar-capillary barrier, thus stimulating the migration of inflammatory cells and lung destruction. MMP-9 has the potential as an early indicator of respiratory failure in COVID-19.	[63, 64]
JAK/STAT	The SARS-CoV-2 infection triggers inflammation via the JAK/STAT pathway, resulting in the recruited pneumocytes, endothelial cells, monocytes, lymphocytes, macrophages, natural killer cells, and dendritic cells progressing towards cytokine storm → production of inflammatory markers and mediation of immune responses via B cell and T cell differentiation. Advanced stage (critically ill COVID-19 patients) → cytokine storm (inflammatory mediators using the JAK/STAT signaling pathway such as IL-6, IFN-γ, result in an influx of macrophages and neutrophils damaging the lung tissue)	[65, 66]
p38 MAPK	Disproportionately upregulated p38 MAPK in SARS-CoV-2 can be a result of infection due to loss of ACE2 activity upon viral entry and by direct viral activation of p38 MAPK. The crucial role of p38 MAPK in the release of proinflammatory cytokines and acute lung injury. p38 MAPK activation may also promote viral entry via ACE2	[67]
ROS	Excessive ROS causes RBC membrane peroxidation that perpetuates neutrophil activation. Exaggerated oxidative stress might be responsible for alveolar damage, thrombosis, and RBC deregulation in COVID-19.	[68]
COX and PGs (PGE-2)	Pro-inflammatory action in COVID-19, PGE2 inhibition can promote the host immune response.	[69]
Ang-2, ICAM-1	Markers of endothelial and/or alveolar epithelial injuries (elevated in COVID-19-related ARDS non-survivors when compared with survivors).	[70]
MIP 1-α	Often increased in COVID-19 patients.	[71]
NO, iNOS	The association between disruption of NO physiology and ARDS development in COVID-19. NO produced by eNOS is compromised (induces alteration in lung parenchyma and coagulopathy), but NO produced by iNOS increases with an effort to fight the virus. Exaggerated NO can generate pro-inflammatory effects.	[72, 73]
TLRs (a subfamily of PRR)	Recognize SARS-CoV-2 in the extracellular milieu or endosomes and mediate the inflammatory signaling that leads to proinflammatory cytokine production. TLR4 is highly expressed during lung injury.	[19, 74]
STAT3, PAI-1	Positive feedback loop between STAT3 and PAI-1 - association with COVID-19; upregulated PAI-1 leads to intravascular thrombi and overproduced PAI-1 binds to TLR4 on macrophages and induces the release of pro-inflammatory cytokines and chemokines. The subsequent activation of innate immune cells within the infected lung leads to the destruction of lung architecture and subsequent hypoxic environment further stimulates PAI-1 production. In addition, acute lung injury activated EGFR and phosphorylation of STAT3. Indeed, DAD also increases PAI-1 levels.	[75]
BRD4	BRD4 is an epigenetic reader of acetylated lysines that plays an important role in epithelial-driven and NF-κB-dependent innate inflammation during viral infection.	[19, 51, 76, 77]
Nrf2	The association between respiratory viral infections, inflammation, and oxidative stress of the epithelium lining cells → activation of Nrf2 to protect cells from oxidative damage and inflammation. The severity of COVID-19 is related to preexisting conditions (impaired immune responses, obesity, or age, which are associated with decreased Nrf2 levels). Nrf2 activation reduces inflammation, restores the cellular redox balance, and facilitates tissue repair.	
PPARγ	Dysfunction in M1 monocytes/macrophages in the innate immune response is characterized by the repression of PPARγ; this is associated with the cytokine storm induced by inflammatory monocytes/macrophages in the SARS-CoV-2-infected lung.	[78]

Abbreviations: ACE2, angiotensin-converting enzyme 2; ALI, acute lung injury; Ang-2, angiopoietin-2; BRD4, bromodomain-containing protein 4; COX, cyclooxygenase; eNOS, endothelial nitric oxide synthase; GM-CSF, granulocyte-macrophage colony-stimulating factor; ICAM-1, intercellular adhesion molecule-1; IFN-γ, interferon-gamma; IL, interleukin; iNOS, inducible nitric oxide synthase; JAK/STAT, Janus kinase/signal transducers and activators of transcription; MIP 1-α, Macrophage inflammatory protein 1-α; MMP, Matrix metalloproteinase; NF-κB, nuclear factor-kappa B; NO, nitric oxide; Nrf2, nuclear factor erythroid-derived 2-related factor 2; p38 MAPK, p38 mitogen-activated protein kinases; PAI-1, plasminogen activator inhibitor-1; PAI-1, plasminogen activator inhibitor-1; PGE2, prostaglandin E2; PGs, prostaglandins; PPARγ, peroxisome proliferator-activated receptor; PRR, pattern recognition receptors; RBC, red blood cell; ROS, reactive oxygen species; TLRs, Toll-like receptors

## Phenolic phytochemicals and phenolic rich herbal medicine against coronavirus-associated lung injury

**Fig. 2:**
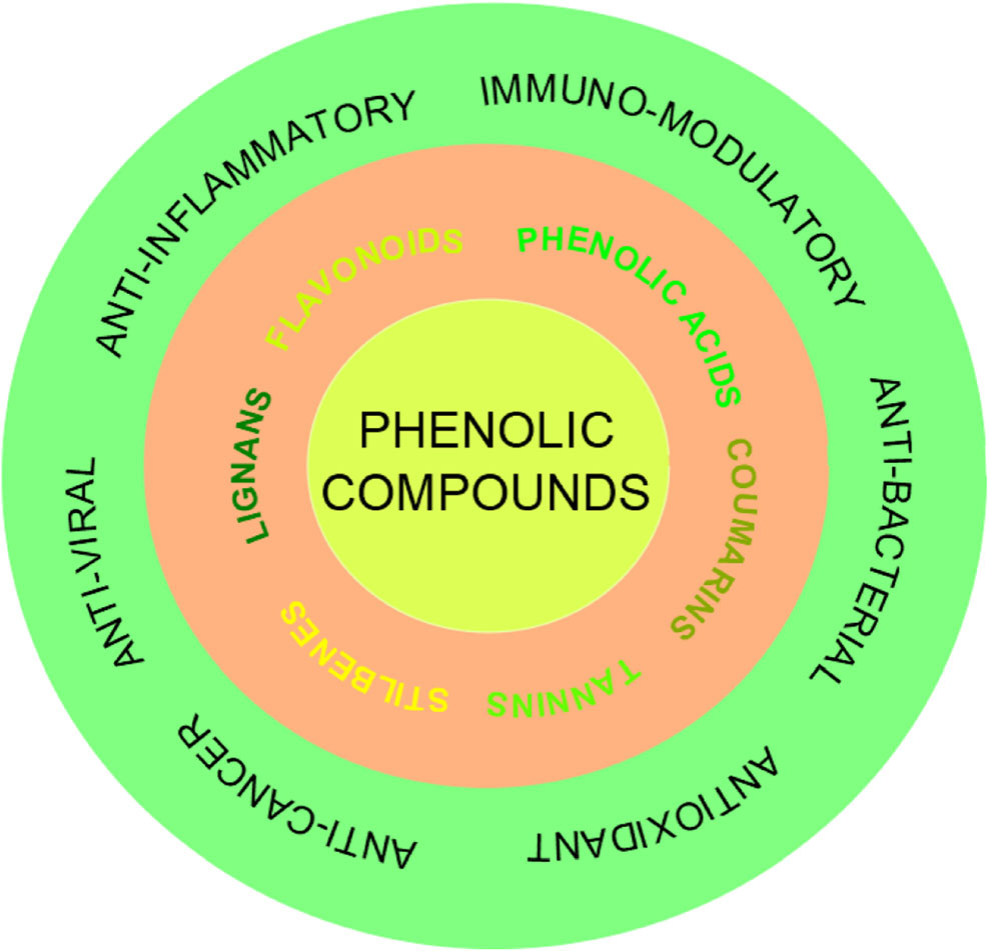
Classification and biological activities of plant phenolic compounds

Phenolic compounds represent the most pronounced plant secondary metabolites [35]. More than 8 000 phenolic compound structures are currently known [79, 80]. Phenolic compounds comprise a heterogenous group of phytochemicals with phenyl rings bearing one or more hydroxyl groups [81]. The most important dietary phenolic compounds include simple phenols, phenolic acids, flavonoids, stilbenes, coumarins, lignans, and tannins [79, 80]. Figure 2 summarizes the classification of phenolic compounds and their biological activities.

This review discusses selected phenolics that occur in plants most frequently and their impact on inflammatory and immunologic pathways deregulated by COVID-19 and in course of the associated lung injury. Phenolic acids are considered the main plant phenolic compounds [80, 82, 83]. Flavonoids represent the largest group of phenolic compounds and comprise more than 6 000 compounds in dietary plants [20, 84–86]. A representative stilbene is a resveratrol that is found mainly in the skin of grapes and wine [79, 87]. Coumarins, which are found in plants such as sweet grass and vanilla grass, constitute another subclass of phenolics; coumarins are also metabolites of microorganisms such as *Streptomyces* and *Aspergillus* species [79]. Curcumin, the main natural polyphenol found in the rhizome of *Curcuma longa*, is also widely known for its biological activities [88–91]. In addition to isolated phenolic compounds, whole plants contain a mixture of various phytochemicals including phenolics in a large extent. The presence of various phytochemicals within plants is considered to contribute to more pronounced pharmacological activity due to additive and synergistic effects [92, 93]. Plants of traditional medicine rich in phenolic phytochemicals represent sources of various bioactive compounds [19, 29, 94]. Despite significant pharmacological activities, the disadvantage of the usage of naturally occurring phytochemicals in vivo is their low water solubility, low gastrointestinal absorption, poor stability in body fluids, rapid metabolism, and rapid clearance [95]. Nevertheless, the ongoing research is intensively identifying novel approaches to increase the bioavailability or promote the effectiveness of targeted delivery of phenolic compounds through the introduction of specific strategies such as nanocarrier systems [19, 96–99]. After all, due to the numerous biological effects including but not limited to antioxidant, anticancer, anti-inflammatory, immunomodulatory activities [83, 84, 93, 100–103], and their important role in managing lung injury by multiple mechanisms [104], phenolics are suggested to possess significant capacity against COVID-19.

### Phenolic acids

Phenolic acids are found in fruit seeds and skin and vegetable leaves [80]. Phenolic acids include hydroxybenzoic acids (HBAs) and hydroxycinnamic acids (HCAs), which are derived from benzoic and cinnamic acid, respectively [82, 83]. HBAs include gallic acid, salicylic acid, ellagic acid, and protocatechuic acid, while major HCAs include p-coumaric acid, caffeic acid, and ferulic acid. Phenolic acids represent pharmacologically active phytochemicals that exert antioxidant, anticancer, anti-inflammatory, or immunomodulatory efficacy [36]. Ellagic acid and gallic acid both demonstrate anti-inflammatory effects through the inhibition of lipopolysaccharide (LPS)-induced NO, PGE-2, and IL-6 production in vitro [105]. LPS-induced inflammation is accepted as a classic inflammatory model [106]. Also, gallic acid inhibits the inflammatory response in macrophages through the blockage of TLR4/NF-κB induced by LPS [107]. In addition, gallic acid has been recently observed to effectively modulate pulmonary inflammation associated with chronic obstructive pulmonary disease (COPD), an inflammatory lung disease, in elastase (ET) and cigarette smoke (CS)-induced mice model demonstrated through the attenuation of pro-inflammatory cytokines (IL-6, TNF-α, IL-1β) and to downregulate gene expression of elevated expression of pro-inflammatory factors such as TNF-α, IL-1β, MIP-2, KC, and GCSF in lungs [108]. Moreover, caffeic acid derivatives show potential for the treatment of ALI demonstrated in a model of mouse primary peritoneal macrophages activated by LPS [109]. Similarly, protocatechuic acid exerts protective effects against LPS-induced ALI in mice and reduces TNF-α and IL-1β by suppressing p38MAPK and NF-κB [110]. Based on well-described anti-inflammatory and immunomodulatory activities, phenolic acids could represent an abundant source of highly effective anti-COVID-19-lung damage agents [111]. Molecular docking, a computational technique to estimate the affinity of association between two molecules (e.g., protein-ligand or protein-protein), [112] is an effective tool for in silico screening in drug discovery [113]. To this end, molecular docking simulations revealed that specific gallic acid derivatives inhibit five nonstructural SARS-CoV-2 proteins [111]. Similarly, the confirmed capacity of caffeic acid derivatives as SARS-CoV-2 inhibitors can support the development of lead structures in COVID-19 therapy or prophylaxis [114]. Moreover, molecular docking has been recently utilized to assess specific biologically active compounds of medicinal plants (such as gallic acid, quercetin, naringin, or capsaicin) in the inhibition of SARS-CoV-2 infection [112]. Lastly, a preliminary report by Tito et al. suggests that a pomegranate peel extract rich in polyphenols, such as gallic acid, ellagic acid, glycosylated derivatives, anthocyanins, and ellagitannins, is a promising source of novel agents against COVID-19 [115].

In conclusion, the biological activities of phenolic acids predetermine them as potentially beneficial compounds against COVID-19-associated complications.

### Flavonoids

Flavonoids represent a group of polyphenolic phytochemicals with potent pharmacological activities. Main flavonoid classes include flavonols, flavones, flavanones, flavanols (the monomer form catechins), isoflavones, flavanonols, and anthocyanidins [20, 84]. Despite antioxidant or anticancer capacity [19, 84, 102], flavonoids are also effective anti-inflammatory and immunomodulatory agents [113]. Various flavonoids (such as luteolin, apigenin, quercetin, rutin, naringin, and naringenin) exert anti-inflammatory effects through the modulation of inflammatory mediators such as TNF-α, IL-1β, IL-6, IL-8, and IFN-γ [116–120]. Other plant-derived flavonoids (flavonoid derivatives and flavonoid glycosides) such as tanshinone IIA, hyperoside, kaempferol, astragalin, isorhamnetin, isovitexin, rhamnazin, morin, troxerutin, engeletin, silibinin, sakuranetin, and 2′*O*-galloylhyperin were reported with the ability to manage acute lung injury by multiple mechanisms of action [104]. Moreover, flavonoids can stimulate the switch of macrophages from pro-inflammatory to anti-inflammatory phenotype [19, 121] and also regulate functions of immunity through the enhancement of the activity of NK cells and cytotoxic T lymphocytes and also through the macrophage functions via modulation of lysosomal activity and the release of NO [19, 120]. Besides, flavonoids can attenuate lung injury as demonstrated by the capacity of baicalein, an active compound of *Scutellaria baicalensis* Georgi belonging to the flavone subclass of flavonoids [84, 102], to reduce histological damage and lung cell apoptosis and inhibit IL-6, IL-1, and TNF-α in a rat model of lung injury induced by myocardial ischemia and reperfusion [122]. Similarly, luteolin attenuated sepsis-induced ALI in mice by suppressing ICAM-1, NF-κB, oxidative stress, and the iNOS pathway [123], and protected against mercuric chloride-induced lung injury in mice by preventing NF-κB activation and activating Akt/Nrf2 [124]. In addition, hesperidin alleviated influenza A-induced lung injury in rats through the inhibition of cytokine production in pulmonary microvascular endothelial cells by suppressing MAPK signaling [125]. As discussed above, the exaggerated immune response caused by SARS-CoV-2 infection can result in a cytokine storm, thrombotic events, ARDS, and multiple organ failure. Cytokine storm-induced lung damage is a direct cause of death from acute COVID-19 [19]. Therefore, the anti-inflammatory and immunomodulatory activities of flavonoids utilizable in COVID-19 are currently largely investigated. The flavonoid-based phytomedicine caflanone binds with high affinity to the spike protein, helicase, and protease sites on the ACE2 receptor used by SARS-CoV-2 to infect cells; moreover, caflanone inhibits the production of cytokines including IL-1β, IL-6, IL-8, Mip-1α, and TNF-α in vitro [113]. In addition, the pharmacological activity of citrus fruits—particularly their flavonoid component hesperidin—has been recently discussed in the context of COVID-19 [126]. Indeed, hesperidin exerts anti-inflammatory effects demonstrated by decreasing IL-33 and TNF-α in mice co-treated with hesperidin and LPS [127]. Apart from their anti-inflammatory and immunomodulatory effects, the effects of flavonoids on lung tissue are also currently evaluated in COVID-19 research. Baicalein exerts a potent pharmacological capacity including antiviral efficacy. Preclinical trials demonstrate the capacity of baicalein to inhibit SARS-CoV-2-induced cell damage in Vero E6 cells. Baicalein also inhibits viral replication and relieves lung tissue lesions in hACE2 transgenic mice infected with SARS-CoV-2. Moreover, baicalein administration improves respiratory function, inhibits inflammatory cell infiltration in the lung, and decreases serum levels of IL-1β and TNF-α in mice with LPS-induced acute lung injury [128]. Another bioactive flavonoid from *Scutellaria baicalensis* Georgi named baicalin has recently been shown to inhibit the replication of SARS-CoV-2 evaluated in Vero and human Calu-3 cells with EC_50_ values of 9.0 and 8.0 μM, respectively. The underlying mechanism was revealed via inhibiting SARS-CoV-2 RNA-Dependent-RNA Polymerase [129]. Another mechanism of action was also disclosed in an in vitro assay via suppressing the activity of SARS-CoV-2 3CLpro (IC_50_ = 6.41 μM), a protease enzyme required for SARS-CoV-2 replication [130]. Furthermore, baicalin in multiple in vivo experiments was noticed to relieve lung impairment as revealed by attenuated myeloperoxidase (MPO) activity, lung edema, and lung histopathologic changes in acute lung injury models caused by various stimuli including some viruses from coronavirus family [131–133]. Baicalin was also found to decrease pulmonary inflammation by down-regulating the expression of pro-inflammatory cytokines TNF-α, IL-1β, IL-6, IL-8, IL-18, IL-23, and matrix metallopeptidase 9 (MMP9) [131]. Moreover, lung oxidative injury was detected to be suppressed by baicalin through decreasing malondialdehyde (MDA) [134]. In an animal study, baicalin (50 mg/kg ) was proved to significantly improve pulmonary function, inflammatory cell infiltration, and cytokine expression (TNF-α, IL-6, and MMP9) [135]. Considering the above-mentioned animal studies, we can conclude that baicalin has a promising application in the treatment of acute lung injury or lung damage that are associated with SARS-CoV-2 post infection.

The catechin (−)-epigallocatechin-3-gallate (EGCG), the main flavonoid compound of green tea, has recently been documented in a comprehensive review article that demonstrated its potential protective effect against SARS-CoV-2 infection via multiple mechanisms of action that target viral or host cell proteins evaluated in various preclinical experiments. The protective effects of EGCG are related to preventing cytokine storm-associated acute lung injury/acute respiratory distress syndrome, thrombosis by inhibiting tissue factors and activating platelets, sepsis by inactivating redox-sensitive high mobility group box 1 (HMGB1), and lung fibrosis via increasing Nrf2 and hindering NF-κB activation [136].

Based on the discussed anti-inflammatory, immunomodulatory, and lung-protective activities of flavonoids and supported by current studies focusing on the models of SARS-CoV-2-induced infection, flavonoids could represent a source of potential phytochemicals in the development of prophylactics or therapeutics against COVID-19.

### Stilbenoids

Stilbenoids are phenolic compounds found in plants, berries, and nuts [137] that share a stilbene backbone structure and differ in the nature and position of their substituents. Stilbenoids are phytoalexins, antimicrobial compounds produced in plants de novo to protect them against infections or toxins; this class of compounds includes gnetol, piceatannol, and resveratrol [138]. Stilbenoids are well-known anti-inflammatory agents that target iNOS, COX, leukotrienes, NF-κB, TNF-α, interleukins [139], or PI3K/Akt [137]. Moreover, stilbenoids exert significant antioxidant and immunomodulatory effects [139, 140] and are potent anti-viral compounds [141]. Resveratrol inhibits the replication of various viruses (dengue, Zika, influenza), including MERS-CoV [142]. Moreover, resveratrol demonstrated a capacity to reduce ALI and inflammation in a murine LPS-induced sepsis model through Sirtuin 1 (Sirt1) regulation, an important regulator of inflammation [143]. Similarly, pre-treatment with 3,5,4′-tri-*O*-acetylresveratrol decreased ALI induced by seawater inhalation through interfering with NF-κB and iNOS pathways followed by decreased NO, TNF-α, and IL-1β [144]. Therefore, stilbenoids may be effective against SARS-CoV-2. Resveratrol shows a capacity to inhibit SARS-CoV-2 infection in Vero cells infected with SARS‐CoV‐2 suggesting its potential role as a novel COVID-19 therapeutic [142]. As discussed above, ARDS is a severe complication of COVID-19 patients that results in almost 40% mortality. However, resveratrol has been suggested to attenuate *Staphylococcal* enterotoxin B (SEB)-induced ARDS. Therefore, Alghetaa et al. have evaluated the effects of resveratrol on the gut and lung microbiota in C3H/HeJ mice with SEB-induced inflammatory cytokines, ARDS, and 100 % mortality. Resveratrol demonstrates the capacity to attenuate ARDS at least partially by altering the gut and lung microbiota, specifically through the induction of beneficial bacteria such as *L. reuteri* [145]. Furthermore, pterostilbene is an additional dimethyl ether and a more stable analog of resveratrol that pharmacologically resembles other stilbenes [146, 147]. Based on a preliminary report by Ellen et al., resveratrol and pterostilbene significantly inhibit SARS-CoV-2 infection in primary human bronchial epithelial cells cultured under ALI conditions [147]. Kobophenol A, a bioactive oligomeric stilbenoid isolated from the *Caragana* genus, has effectively inhibited the infectivity of SARS-CoV-2 (in vitro) with an EC_50_ of 71.6 μM. The mechanism was unveiled by blocking the interaction between the ACE2 receptor and S1-RBD in vitro with an IC_50_ of 1.81 μM. The results were also confirmed by molecular docking and molecular dynamic simulation studies [148]. Besides, Kobophenol A has previously been described to possess anti-inflammatory and anti-oxidant activities [149] and therefore might prevent lung injury linked with SARS-CoV-2 infection as well as other inflammatory diseases and cancer.

In conclusion, the potential effects of stilbenoids supported by already proven anti-inflammatory, immunomodulatory, and anti-viral efficacy and initial results from SARS-CoV-2 research require further evaluation under COVID-19 conditions.

### Coumarins

Coumarins are natural compounds found in plants, fungi, and bacteria [150]. Coumarins shows potent anticancer, antibacterial, antifungal, antioxidant, anti-inflammatory, antithrombotic, and antiviral activities [151, 152]. Significant anti-inflammatory effects of sesquiterpene coumarins from *Ferula fukanensis* was demonstrated through the inhibition of NO, iNOS, IL-6, and TNFα gene expression in murine macrophage-like cell line RAW264.7 activated by LPS and recombinant mouse IFN-γ [153]. Therefore, coumarins could exert also a potential efficacy against COVID-19. Coumarin derivatives reveal effectiveness as potential inhibitors of the enzymes essential for SARS-CoV-2 viability in silico [154]. Another study aiming to identify selective antiviral agents for the management of COVID-19 pathologies has demonstrated a capacity of coumarin-24 to be effectively used against COVID-19 infection [155]. Most importantly, in silico screening of natural products isolated from Mexican Herbal Medicines reveals the ability of coumarin cichoriin to reach an acceptable level in plasma and high lung levels, while these results suggest its potential as a novel therapeutic tool against COVID-19 [156]. Due to the significant potential of coumarins against SARS-CoV-2, Chidambaram et al. have recently evaluated the possibility of synthesis of novel coumarin analogues to identify drugs against COVID-19 [157].

Therefore, and similar to other members of plant phenolic compounds, coumarins could also represent a rich and effective source of bioactive compounds targeting infection induced by SARS-CoV-2.

### Traditional herbal medicine rich in phenolics protecting against SARS-CoV-2-induced lung damage

Notably, traditional herbal medicine such as traditional Chinese medicine, with its main active constituents including to a large extent phenolic compounds, could also exert significant activity against lung damage and associated inflammatory and immunomodulatory deregulations observed in COVID-19 [156, 158]. Immune dysfunction is essential for COVID-19 progression; therefore, the administration of phytochemicals or herbal medicines containing certain compounds with antimicrobial, antiviral, anti-inflammatory, and immune-modulatory effects have great potential as effective prophylactic and therapeutic agents against SARS-CoV-2 [159]. Indeed, the blockage of the cytokine storm can represent an effective tool against SARS-CoV-2 [159]. Chinese herbal medicine *Arenaria kansuensis* is known for its antiviral activity has been long used to treat pulmonary disease is suggested to exert beneficial effects also against COVID-19. Therefore, Cui et al. have recently evaluated the protective capacity of *Arenaria kansuensis* ethanol extract (AE) on pulmonary fibrosis in paraquat (PQ)-induced pulmonary fibrosis animal models. The study results reveal improved destruction degree of lung tissue structure with increasing AE dosage, reduced collagen deposition in lung interstitium, and reduced degree of inflammatory infiltration and inflammatory cytokines; indeed, the protective effect of AE on pulmonary fibrosis was partly due to activation of Nrf2 pathway and the inhibition of NF-kB/TGF-β1/Smad2/3 pathway [160]. Another herbal therapy, Shufeng Jiedu has been also suggested as a promising drug for the treatment of COVID-19 demonstrated in the HCoV-229E mice model of lung index. Shufeng Jiedu decreased the viral load in the lung, attenuated cytokine release, and increased T- and B-lymphocytes. The authors conclude that Shufeng Jiedu significantly downregulates the inflammatory factors IL-6, IL-10, TNF-α, and IFN-γ in the lung and increases CD4+ and CD8+ cells in the blood compared to the model group. In addition, ShufengJiedu can reduce NF-κB activity. Moreover, ShufengJiedu constituents quercetin, wogonin, and polydatin bind directly to the main protease (Mpro) of SARS-CoV-2. Therefore, ShufengJiedu is a promising drug against COVID-19; however, further validation in clinical trials is needed [161].

Moreover, a case report recently reported a 61-year-old female COVID-19 patient whose lung inflammatory exudate, pulmonary fibrosis, and quality of life significantly improved after oral treatment with traditional Chinese medicine decoction alone [162]. Plant extracts are complex multicomponent mixtures. Indeed, fingerprinting and chemical profiling are essential for the acceptance and quality control of traditional herbal medicines. Divya-Swasari-Vati (DSV) is a calcium-containing herbal medicine with a long history of use against respiratory infections. DSV is also used to control COVID-19-associated respiratory symptoms [159]. DSV exerts its pharmacological activity against SARS-CoV-2-induced inflammation in a humanized zebrafish model through the amelioration of the inflammation induced by SARS-CoV-2 spike protein demonstrated by the blockage of pro-inflammatory IL-6 and TNFα cytokine surge [163]. Indeed, high-performance liquid chromatography–diode array detection (HPLC-DAD) has been recently used to estimate the levels of several phytochemicals, including but not restricted to phenolics (gallic acid, protocatechuic acid, ellagic acid, coumarin, cinnamic acid, glycyrrhizin, eugenol, 6-gingerol, piperine, methyl gallate, and glabridin), in five batches of DSV [159].

Traditional herbal medicine, which is composed of specific plants containing a mixture of various phytochemicals, has a long history of its applicability to maintaining human health. Therefore, such mixtures of various biological active phytochemicals are suggested to be significantly effective against COVID-19 while this efficacy has been at least partly observed also in a clinical setting.

### Extended polyphenolic compounds

Curcumin (diferuloylmethane) is a yellow pigment and natural polyphenol present in the turmeric spice (*Curcuma longa*) [95, 164]. Curcumin exerts various pharmacological activities, including antioxidant, anticancer, anti-inflammatory, and anti-infective. Curcumin affects various signaling molecules associated with the inflammatory processes, such as TNF-α, IL-1β, NF-κB, COX-2, and iNOS [165, 166]. Curcumin was observed to regulate the differentiation of naïve CD4+T cells and activate IL-10 immune modulation against acute lung injury in mice and thus alleviated lung injury and suppressed uncontrolled inflammation [167]. The evaluation of the effects of curcumin on ALI revealed suppressive activity on EGFR and proliferative protein Ki67 in ALI and lung fibrosis in vitro and in vivo [168].

Similarly, direct pulmonary delivery of solubilized curcumin reduced injury, inflammation, and mortality in a mouse model of lethal pneumonia [169]. Based on the above discussed biological activities, curcumin is suggested as a potential option against COVID-19 due to its capacity to affect not only the viral entrance, encapsulation, and replication but also various signaling cascades of inflammation [96]. EGYVIR is an immunomodulatory herbal extract composed of black pepper extract and curcumin extract. EGYVIR has revealed effectiveness against SARS-CoV-2 in vitro through modulating NF-kβ/TNFα/IL-6 during the infection process. Also, EGYVIR antagonizes NF-κB pathway in silico and in vitro and has the potential to hinder the release of IL-6 and TNFα, thus decreasing the production of elements associated with the cytokine storm [170]. Moreover, Noor et al. recently evaluated the immunomodulatory and anti-cytokine therapeutic potential of curcumin and its derivatives targeted against COVID-19 immunological human host receptors, i.e., ACE2, IL-1β, IL-6, TNF-α, and protease-activated receptor (PAR)-1 to prevent viral infection and control overproduction of early clinical responses. Eventually, computational modeling demonstrates the immunomodulatory and anticytokine therapeutic potential of hydrazinocurcumin against COVID-19; however, further in vivo investigations are needed to confirm hydrazinocurcumin as a COVID-19 drug [171]. Despite significant pharmacological activities, the disadvantage of the usage of natural phytochemicals such as curcumin includes its low bioavailability and rapid metabolism [95]. The study conducted on COVID-19 patients reveals a significant decrease in Th17 cells, Th17 cell-related factors, and levels of Th17 cell-related cytokines in mild and severe COVID-19 patients treated by nano-curcumin compared to the placebo group thus demonstrating the potential of curcumin to improve COVID-19 patient’s inflammatory condition [172]. Moreover, an evaluation of nano-curcumin oral formulation effectiveness in hospitalized patients with mild-to-moderate COVID-19 has revealed its capacity to significantly improve recovery time in hospitalized patients [96]. In addition, Miryan et al. have introduced a protocol for a randomized controlled trial to evaluate the capacity of curcumin-piperine co-supplementation on disease duration, severity, and clinical symptoms, and inflammatory mediators in COVID-19 patients [173].

Last but not least, specific Nrf2-interacting nutrients reveals a promising potential against COVID-19 [174]. Genetic or pharmacological Nrf2 activation is associated with anti-inflammatory and antiviral efficacy in various pathologies while targeting specific cysteine receptors within KEAP1 is considered as the most relevant mechanism of such action. Although the potential effects of Nrf2 inducers for the reduction of oxidative stress and inflammation in COVID-19 is not fully elucidated yet, it can be hypothesized that phenolic compounds could reduce COVID-19 severity via the activation of Nrf2 and subsequentmodulation of inflammatory and immune processes [51, 77]. Nrf2-interacting nutrients (berberine, curcumin, epigallocatechin gallate, genistein, quercetin, resveratrol, sulforaphane) reduce insulin resistance, endothelial damage, lung injury, and cytokine storm and act on several mechanisms such as mTOR, PPARγ, NF-κB, ERK, etc. Nrf2-interacting nutrients can promote the mitigation of COVID-19 severity through the endoplasmic reticulum stress, ACE-angiotensin-II-AT1R axis (AT1R). Indeed, geographical areas with very low COVID-19 mortality are those with the lowest prevalence of obesity and intake of fermented food associated with Nrf2 activation (sub-Saharan Africa, Asia, Central Europe) [174].

In conclusion, apart from the most known classes of phenolics, other plant species (*Curcuma longa*) or specific phytochemicals characterized as Nrf2-interacting nutrients of the phenolic structure shows a potential to be effective modulators of inflammatory and immune pathways associated with COVID-19 and related organ damage.

Table 2 provides a detailed overview of current research on the anti-inflammatory and immunomodulatory properties and the overview of the most current in silico, molecular docking screening, and clinical trials evaluating the effects of plant phenolics that can be utilizable also in the search for novel treatment modalities against lung damage induced by COVID-19.

**Table 2:** Potential actions of specific plant phenolics against lung damage induced by SARS-CoV-2

Phenolic compound	Study details	Mechanisms	Effects	Reference
Phenolic acids				
Ellagic acid and gallic acid	RAW264.7 cells	Inhibition of LPS-induced NO, PGE-2, IL-6	Anti-inflammatory	[105]
Gallic acid	RAW264.7 macrophages	Blockage of TLR4/NF-κB induced by LPS	Anti-inflammatory	[107]
Gallic acid	ET- and CS-induced murine model	ET: decrease in IL-6, TNF-α, IL-1β CS: decrease in TNF-α and the inflammatory chemokines MIP-2 and KC.	Modulation of COPD-associated pulmonary inflammation	[108]
Caffeic acid derivatives	Murine primary peritoneal macrophages activated by LPS	Prevented formation of LPS/MD2/TLR4	Anti-inflammatory (potential for ALI treatment)	[109]
Protocatechuic acid	LPS-induced ALI in a murine model	Reduced TNF-α and IL-1β mediated through suppressed p38MAPK and NF-κB	Protective effects against ALI	[110]
Gallic acid derivatives	Molecular docking	Inhibitory effects against five non-structural SARS-CoV-2 proteins	Potential against SARS-CoV-2	[111]
Bioactive compounds of medicinal plants	Molecular docking	Potential inhibition of SARS-CoV-2 infection		[112]
Pomegranate peel extract rich in polyphenols	Preliminary report	A promising source of novel agents against COVID-19		[115]
Flavonoids				
Baicalein	Rat model of lung injury induced by myocardial ischemia and reperfusion	Reduced histological damage and apoptosis in the lung; downregulated IL-6, IL-1, and TNF-α	Attenuation of lung injury	[122]
Luteolin	Murine model	Suppressed ICAM-1, NF-κB, oxidative stress, iNOS; Reduced IL-6 and IL-1β in lung tissue	Attenuation of sepsis-induced ALI	[123]
Luteolin	Murine model	Prevented NF-κB activation and activated Akt/Nrf2	Protection against mercuric chloride-induced lung injury	[124]
Hesperidin	Rat model of lung injury induced by influenza A	Inhibited cytokine production in pulmonary microvascular endothelial cells by suppressing MAPK	Alleviated lung injury	[125]
Flavonoid-based phytomedicine *caflanone*	Molecular docking; in vitro (Hcov-OC43 human coronavirus)	Capacity to inhibit the production of cytokines including IL-1β, IL-6, IL-8, Mip-1α, TNF-α High affinity for spike protein, helicase, and protease ACE2 sites	Potential against SARS-CoV-2	[113]
Hesperidin	Mice co-treated with hesperidin and LPS	Decreased IL-33 and TNF-α	Anti-inflammatory - potential against SARS-CoV-2	[127]
	Vero E6 cells, hACE2 transgenic mice infected with SARS-CoV-2; LPS-induced acute lung injury of mice	Vero E6 cells: inhibited SARS-CoV-2-induced cell damage hACE2 transgenic mice infected with SARS-CoV-2: LPS-induced acute lung injury of mice: inhibited viral replication and relieved the lung tissue; improved respiratory function, inhibited inflammatory cell infiltration in the lung, decreased IL-1β and TNF-α		[128]
Stilbenoids				
Resveratrol	Murine LPS-induced sepsis model	Sirt1 activation	Reduction of ALI and inflammation	[143]
3,5,4′-Tri-*O*-acetylresveratrol	Model of ALI induced by seawater inhalation in rats	Inhibited NF-κB and iNOS followed by decreased NO, TNF-α, IL-1β	Reduction of ALI	[144]
Resveratrol	Vero cells infected with SARS‐CoV‐2	Capacity to inhibit SARS-CoV-2 infection (potential role as a novel COVID-19 therapeutic)		[142]
Resveratrol	Model of C3H/HeJ mice with SEB-induced inflammatory cytokines, ARDS, and 100% mortality	Altered gut and lung microbiota (*L. reuteri* induction)	ARDS attenuation	[145]
Resveratrol and pterostilbene	Primary human bronchial epithelial cells cultured under ALI conditions	Inhibited SARS-CoV-2 infection		[147]
Coumarins				
Sesquiterpene coumarins from *Ferulafukanensis*	Murine macrophage-like cell line RAW264.7 activated by LPS and recombinant mouse IFN-γ	Inhibited NO, iNOS, IL-6, and TNF-α gene expression	Anti-inflammatory	[153]
Cichoriin	In silico	Capacity to reach high lung levels	Suggested potential as a novel COVID-19 therapeutic	[156]
Traditional herbal medicine				
AE	PQ-induced pulmonary fibrosis animal model	Nrf2 activation and NF-κB/TGF-β1/Smad2/3 inhibition	Protective capacity against pulmonary fibrosis (lessened the destruction of lung tissue, reduced collagen deposition (lung interstitium), inflammatory infiltration, and inflammatory cytokines)	[160]
ShufengJiedu	HCoV-229E murine model of lung index, viral load in the lung, the release of cytokines, and T- and B-lymphocytes	Decreased IL-6, IL-10, TNF-α, IFN-γ in the lung and increased CD4+ and CD8+ cells in the blood; reduced NF-κB activity	Anti-inflammatory (potential against SARS-CoV-2)	[161]
Oral treatment with Traditional Chinese medicine decoction without any other drugs	Case report (61-year-old female with COVID-19)	Improved lung inflammatory exudate, pulmonary fibrosis, and quality of life		[162]
DSV	Model of SARS-CoV-2 induced inflammation in the humanized zebrafish model	Blocked IL-6 and TNF-α cytokine surge	Anti-inflammatory (pharmacological activity against SARS-CoV-2 induced inflammation)	[163]
	Murine ALI model	Regulated the differentiation of naïve CD4+T cells and activated IL-10 immune modulation	Alleviated lung injury and suppressed uncontrolled inflammation	[167]
Curcumin				
Curcumin	Bleomycin-induced basal alveolar epithelial cells and C57BL/6 mice	Suppressed EGFR, Ki67, and lung fibrosis	Alleviated lung fibrosis	[168]
Solubilized curcumin (direct pulmonary delivery)	Murine model of lethal pneumonia (C57BL/6 mice inoculated with a lethal dose of *Klebsiella pneumoniae*)	Decreased TNF-α, IFN-β, NF-κB	Reduced injury, inflammation, and mortality	[169]
EGYVIR (pepper and curcumin extract)	*In silico*, *in vitro*	Modulated NF-κB/TNF-α/IL-6 (downregulated IL-6 and TNF-α)	Potential to decrease cytokine storm	[170]
Hydrazinocurcumin	Computational modeling		Immunomodulatory and anticytokine therapeutic potential	[171]
Nanocurcumin	Randomized, double‐blind, placebo‐controlled–mild (*n* = 40) and severe (*n* = 40) COVID-19 patients	Decreased Th17 cells, Th17 cell-related factors, and levels of Th17 cell-related cytokines	Potential to improve COVID-19 patients’ inflammatory conditions	[172]
Nanocurcumin oral formulation	Open-label nonrandomized clinical trial Hospitalized patients with mild-to-moderate COVID-19, nano-curcumin (*n* = 21) and control (*n* = 20) group	Improved recovery time		[96]
Curcumin-piperine co-supplementation	Randomized controlled trial	Introduced protocol for the trial (evaluation of disease duration, severity, and clinical symptoms)		[173]
Nrf2-interacting nutrients				
Nrf2-interacting nutrients	Modulation of endoplasmic reticulum stress and the theACE-angiotensin-II-AT1R axis		Capacity to mitigate COVID-19 severity	[174]

Abbreviations: ACE, angiotensin-converting enzyme; AE, *Arenaria kansuensis* ethanol extract; Akt, protein kinase B; ALI, acute lung injury; AT1R, angiotensin II receptor type 1; COPD, chronic obstructive pulmonary disease; COX-2, cyclooxygenase-2; CS, cigarette smoke; DSV, Divya-Swasari-Vati; EGFR, epidermal growth factor receptor; ET, elastase; ICAM-1, intercellular adhesion molecule; IFN-γ, interferon γ; IL, interleukin; iNOS, inducible nitric oxide synthase; LPS, lipopolysaccharide; MAPK, mitogen-activated protein kinase; MD2, myeloid differentiation protein 2; Mip-1α, macrophage inflammatory protein 1α; NF-κB, nuclear factor-κB; NO, nitric oxide; Nrf2, nuclear factor erythroid 2-related factor 2; PGE-2, prostaglandin E2; PQ, paraquat; TGF-β1, transforming growth factor β1; TLR4, Toll-like receptor 4

## Conclusions and future perspectives in the framework of 3PM

Naturally occurring plant substances have long been considered as effective helpers in maintaining good physical and mental shape against health adverse effects of different origin. Phytochemicals—both native ones in the intact plants and their extracts or pharmacologic derivatives show a wide range of health-protective systemic effects. To this end, anti-inflammatory, immunomodulatory, and organ-protective effects of plant phenolic compounds are promising for protective treatments under the COVID-19 pandemic condition.

More specifically, plant phenolics (phenolic acids, flavonoids, coumarins, stilbenoids) pleiotropic activity modulates inflammatory mediators (IL-6, TNF-α), oxidative stress, and specific signaling cascades (NF-κB, Nrf2 among others) that is utilized by searching for effective compounds against COVID-19 infection and cascading complications such as cytokine storm, systemic inflammation and associated organ damage. Corresponding protective effects are evidence-based as demonstrated in the current paper.

*Caution!* Although in preclinical studies natural phytochemicals demonstrate evidence-based effects against SARS-CoV-2 infection and organ damage, the consumption of unproven and unapproved products is a great health risk to be avoided by consultating accrediated specialists in the area.

**Fig. 3:**
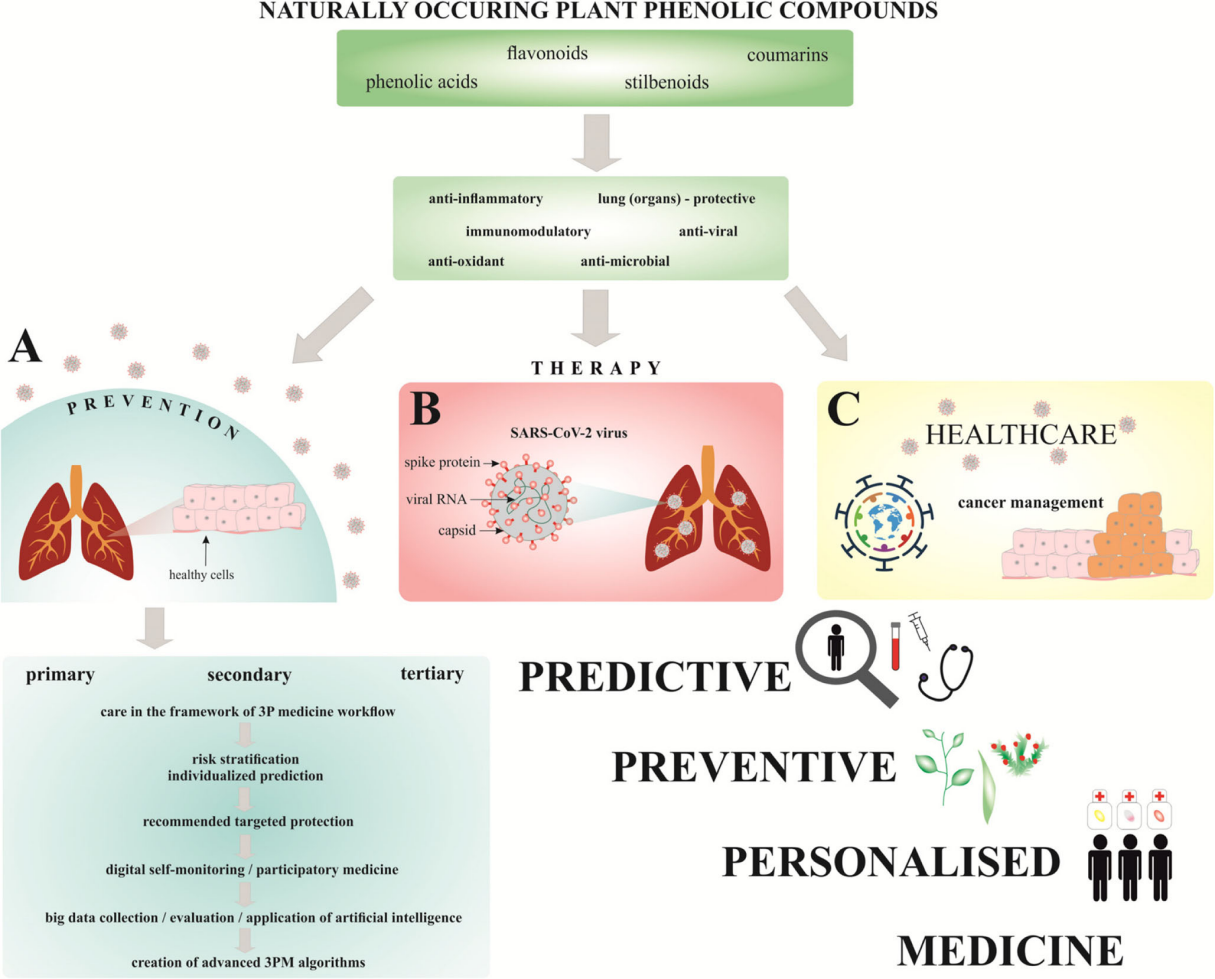
Application of plant phenolics to mitigate COVID-19-associated systemic damage in the framework of 3PM

In order to promote clinically relevant research and facilitate cost-effective treatments in the close future, Table 3 summarizes prominent examples of stratified patient groups, corresponding risks and mitigating measures presenting evidence-based molecular and cellualr mechanisms as well as expected health effects. To mitigate corresponding risks in primary, secondary and tertiary care, phytoprotection has to be targeted to the stratified patients groups avoiding potential negative side effects and increasing the oveall treatment efficacy. Figure 3 summarizes general concepts in the frame-work of predictive, preventive, and personalized medicine (3PM) [175–177].

**Table 3:** Prominent examples on stratified groups of risk for targeted COVID-19 management increasing efficiacy of the plant phenolics appplication

A					B								
Stratified risk	Targeted protection/risk mitigation	Comments on PPPM primary/secondary/tertiary care	Ref	Phytosubstance	Effects	Proposed mechanisms of action	Plants	Form of natural availability (adapted medication)	Study	Ref	Expected effects of plant phenolics to be explored	Available evidence	Ref
Sleep deprivation in COVID-19 patients	Immune system functionality (T-Help cells)	Poor sleep quality associated with decreased immune system functionality in COVID-19 patients, improved sleep quality cold improve recovery and individual outcomes (patient stratification, individualised prediction, targeted prevention of high risk persons)	[9]	Flavanones, flavones, phenolic acids, lignans	Decreased likelihood of inadequate sleep quality	Improved resilence after sleep deprivation, prolonged sleep time, shortening sleep latency	Flavonoids-rich food	Dietary polyphenols	1936 adults	[178]	Improved sleep quality to promote immune responses	Inadequate sleep impairs immune system; polyphenols improve quality of sleep	[9, 179, 180]
Sleep quality in COVID-19 healthcare givers	Poor sleep quality, insomnia, mental health risks (stress, predisposition to depression, anxiety)	Social and psychological support considered as preventive strategy; consideration of sleep quality and psychiatric symptoms in healthcare givers treating COVID-19 patients		Polyphenols (verbascoside and flavone diglucuronides such as luteoline‐7‐diglucuronide)	Complementary option for improving sleep quality and reducing insomnia severity		*Aloysia citriodora*	Lemon verbena (*A. citriodora* syrup)		[181]			
Periodontitis	Systemic inflammation and pneumonia development, increased risk of intensive care unit admission of COVID-19 patients	The need of active participation of patients in primary and secondary care—essential for PPPM approach	[9, 10]	Flavonoids	Improved periodontal health	Decresed IL-1β (marker of periodontitis)	Flavonoid rich diet	Flavonoids from food and beverages	43 post‐scaling and root planing patients	[182]	Anti-inflammatory	Periodontal health, anti-inflammatory effects	[183, 184]
Active smoking or history of smoking	Immune responses, respiratory function (severe COVID-19)	The evaluation of smoking/smoking history essential to consider individual risk of COVID-19 (primary care) or progression	[11, 185, 186]	Flavonoids	Improved inflammatory status in healthy smokers	Decreased sICAM-1	Grapes	Concord grape juice	26 healthy smokers	[187]	Anti-inflammatory, improved immune responses,	Immune responses, lung protective effects, modulation of inflammation	[108, 112, 113, 169]
Chronic lung diseases (COPD)	Association of COPD and severe COVID-19 (due to, e.g., higher ACE2 expression)	The evaluation of chronic lung diseases, such as COPD, is essential to precisely recognize individuals at higher risk of SARS-CoV-2 infection and/or severe diseases course	[12]	Flavonoids (resveratrol)	Improved inflammatory status	Inhibited inflammatory cytokine release	Fruits and vegetables	Red wine (wine extract)	15 COPD patients	[188]			
Chronic inflammation (asthma)	The manifestation of COVID-19 in patients with asthma associated with age (comorbidities—high blood pressure, diabetes, obesity, dyslipidemia)	Required stratification of patients at increased risk of COVID-19 related to occurrence of asthma, especially in the elderly	[13]	Flavonoids	Decresaed incidence of asthma (age-related)	Less common asthma	Flavonoid-rich food	Apples, red wine	9,709 individuals	[189]	Asthma in the elderly as risk factor; plant phenolics against asthma nd chronic inflammation	Inflammation, allergies, immunity	[13, 190, 191]
Metabolic ill patients with obesity	Obesity associated with COVID-19 comorbidities; metabolic ill patients with obesity—increased IL-6, positive correlation with CRP and waist-hip-ratio	Required evaluation and early identification of the risk of hyperinflammation essential for adequate management of COVID-19	[14]	Flavonoids (hesperidin)	Anti-inflammatory effects	Reduced high-sensitivity CRP	Citrus fruit	Capsules (500 mg hesperidin)	28 individuals with metabolic syndrome	[192]	Inflammation in obese individuals	Metabolic inflammation and oxidative stress (obesity),	[43, 193, 194]
Vascular function	Lower systemic vascular function and higher arterial stiffness evident weeks after testing positive for SARS-CoV-2 (young adults)	Required consideration of risk assessment of post-COVID-19 complications and associated appropriate management	[15]	Polyphenols	Cardiovascular protection	Decreased stiffness index, decreased blood pressure	Black tea	Beverage (tea)	19 essential untreated hypertensive patients	[195]	Cardiovascular functions, protection against cardiovascular damage	Cardiovascular protection, inflammation, anti-thrombotic effects	[136, 151, 152, 196, 197]
	Arterial stiffness and admission systolic blood pressure < 120 mm Hg suggested as independent prognostic value for all-cause mortality in patients with COVID-19 requiring hospitalization	Required consideration of specific conditions, e.g. arterial stiffness or blood pressure to predict individual COVID-19 prognosis	[16]										
Prostate cancer	Increased risk of SARS-CoV-2 infection; patients receiving androgen-deprivation therapy partially protected from infection	The evaluation of overall health conditions (e.g., cancer) and cancer treatment in specific groups of cancer patients is essential for precise, individualised, and targeted COVID-19 therapy and protection against poor outcome	[17]	Lycopene, isoflavones	Anti-cancer and immunomodulatory effects	Delayed progression	Soya isoflavone	Soya	73 prostate cancer patients	[198–200]	Modulation of cancer patients immune responses, protection against poor outcome	Modulation of immunity, inflammation, prevention	[19, 113, 201, 202]
	Protective role of androgen-deprivation therapy against SARS-CoV-2 infections seems to be lower in metastatic prostate cancer patients		[203]	Flavonoids		Modulation of immune system, tumor microenvironment	Dietary polyphenols/polyphenols (silibinin)		In vitro (prostate cancer cell lines)				
Hypoxemia	Association with in-hospital mortality	The evaluation of possible association between hypoxemia and COVID-19 mortality could improve clinical management of COVID-19 patients	[204, 205]	Flavonoids (breviscapine, chrysin)	Improved lung tissue affected by hypoxemia	Improved hypoxia-induced hypercoagulable state; alleviated hypoxia	Breviscapine (crude extract of several flavonoids of *Erigeron breviscapus*); chrysin		In vivo (rat models)	[206–208]	Hypoxemia, hypoxia	Targeting hypoxemia; mitigating pulmonary fibrosis (alleviating local tissue hypoxia)	[207–209]

Abbreviations: ACE2, angiotensin-converting enzyme 2; COPD, chronic obstructive pulmonary disease; IL, interleukin; PPPM, predictive, preventive and personalized medicine; sICAM-1, soluble intercellular adhesion molecule-1

## Data Availability

Not applicable.
